# Evaluating the Educational Impact of 3D-Printed Models in Cervical Laminoplasty Training: A Survey-Based Study

**DOI:** 10.7759/cureus.79021

**Published:** 2025-02-14

**Authors:** Manuel De Jesus Encarnacion Ramirez, Carlos Salvador Ovalle Torres, Andreina Rosario Rosario, Gervith Reyes Soto, Carlos Castillo-Rangel, Carlos Castillo Sorian, Francisco Castañeda Aguayo, Nasser M F El-Ghandour, Vladimir Nikolenko, Tshiunza Mpoyi Cherubin

**Affiliations:** 1 Department of Neurosurgery, Peoples' Friendship University of Russia, Moscow, RUS; 2 Department of Human Anatomy and Histology, N.V. Sklifosovsky Institute of Clinical Medicine, Moscow, RUS; 3 Neurosurgical Oncology, Mexico National Cancer Institute, Tlalpan, MEX; 4 Abnormal Movements and Neurodegenerative Diseases Unit (UMANO), National Autonomous University of Mexico, General Hospital, Durango, MEX; 5 Medicine, Autonomous University of Santo Domingo (UASD), Santo Domingo, DOM; 6 Neurosurgery, ISSSTE Regional Hospital October 1, Mexico City, MEX; 7 Department of Neurosurgery, Tecnológico de Monterrey Campus Estado de México, Mexico City, MEX; 8 Neurosurgery, Centro Médico Nacional 20 de Noviembre, Mexico City, MEX; 9 Department of Neurosurgery, Faculty of Medicine, Cairo University, Cairo, EGY; 10 Branch of the Human Anatomy and Histology, N.V. Sklifosovsky Institute of Clinical Medicine, Moscow, RUS; 11 Department of Neurosurgery, Clinique Ngaliema, Kinshasa, COD

**Keywords:** 3d printed, cervical, spine surgery, training, vertebral laminoplasty

## Abstract

Background and objective

The complexities of spinal surgery, particularly the intricacies of cervical pathology, demand precision and expertise in surgical interventions. Cervical laminoplasty is a procedure that requires meticulous execution and a profound understanding of delicate anatomical structures. Recognizing the limitations of traditional training methods, this study highlights the transformative impact of integrating 3D modeling and printing technologies into medical education. These technologies provide an immersive, interactive, and highly detailed training platform, enabling aspiring surgeons to visualize, dissect, and practice procedures in a risk-free environment. Beyond education, 3D models enhance patient-doctor communication, enable precise preoperative planning, facilitate custom implant design, and support a personalized approach to spinal surgery. Collectively, these advancements hold the promise of reducing surgical errors and improving outcomes.

Materials and methods

Thirty-eight participants, including neurosurgeons, residents, and medical doctors, were enrolled in this study. High-resolution CT scans, obtained with informed consent to ensure confidentiality and ethical compliance, were used to create the 3D models. These models, printed with polylactic acid (PLA) filament and refined through post-processing, achieved high anatomical accuracy and quality. The training program combined lectures, live demonstrations, and hands-on sessions with 3D models. Participants' experiences and perceptions were evaluated through a survey, focusing on the models' utility and realism in advancing surgical skills.

Results

The participants overwhelmingly praised the 3D models for their utility in helping to understand cervical laminoplasty concepts and enhancing their learning compared to traditional methods. The models were particularly valued for their accurate representation of anatomical structures and improved visualization of surgical steps. Notably, 81.6% of participants found the models extremely beneficial in planning surgical approaches. The survey results unanimously highlighted the transformative potential of 3D models in medical education. Participants strongly recommended their integration into training programs and preoperative planning processes, emphasizing their ability to elevate the learning experience and improve surgical preparedness.

Conclusions

Our findings show that 3D modeling significantly enhances training in cervical laminoplasty by providing superior learning tools and improving anatomical visualization compared to conventional methods. The unanimous endorsement from participants underscores the adaptability and precision of 3D models in medical education and preoperative planning. As an indispensable resource in modern medical training, these models represent a pivotal advancement in preparing surgeons for the complexities of spinal surgery.

## Introduction

The intricate nature of spinal surgery demands a seamless blend of theoretical expertise, practical skill, and a profound understanding of spinal pathologies. Among these, cervical pathologies, such as cervical myelopathy and radiculopathy, stand out due to their complexity and the precision required for successful surgical intervention. These conditions, often characterized by neural compression leading to pain, numbness, and functional impairments, frequently necessitate surgical redress to restore neurological function and improve the quality of life of patients [1-4].

Cervical laminoplasty is a well-established surgical treatment for cervical myelopathy and radiculopathy. This procedure aims to alleviate spinal cord and nerve root compression by reshaping or repositioning the laminae, thereby creating additional space for the spinal cord without requiring fusion. Techniques such as Hirabayashi's open-door laminoplasty and Kurokawa's double-door approach have solidified the role of this method in addressing these pathologies [5-8]. The ability to preserve spinal mobility while effectively relieving compression underscores the importance of cervical laminoplasty. However, the procedure’s success hinges on meticulous surgical precision and an in-depth understanding of cervical anatomy. The challenges of this procedure, coupled with the limitations of traditional training modalities, have paved the way for transformative advancements in surgical education [9].

Traditional training methods, relying heavily on theoretical instruction, cadaveric dissections, and observational learning, often fail to provide the real-world, hands-on experience necessary for mastering spinal surgeries like cervical laminoplasty. Cadaveric models, while valuable, lack the dynamic feedback and anatomical variability of living patients. This gap between theoretical knowledge and practical application can hinder the development of essential surgical skills [10-12]. 3D printing technology addresses these limitations by creating highly accurate, patient-specific anatomical models from imaging data such as CT or MRI scans. These models allow surgeons to visualize, manipulate, and practice surgical techniques in a risk-free, realistic environment. The tactile feedback and three-dimensional perspective provided by these models foster an unparalleled understanding of spinal anatomy and surgical steps [10,13,14].

The interactive and detailed nature of 3D models creates an ideal platform for both training and surgical planning. For trainees, these models provide the opportunity to perform mock surgeries, practice intricate maneuvers, and develop a deep spatial understanding of cervical anatomy. This hands-on experience helps bridge the gap between theoretical knowledge and clinical practice, significantly improving proficiency and confidence [15-16]. For experienced surgeons, 3D models serve as invaluable tools for preoperative planning. They enable detailed visualization of patient-specific anatomy, allowing surgeons to anticipate potential challenges and refine their surgical strategies accordingly. This level of preparation not only enhances surgical precision but also reduces the likelihood of complications, ultimately improving patient outcomes [10,17-18].

Another transformative application of 3D printing lies in the design and testing of custom implants. Unlike traditional "one-size-fits-all" implants, 3D printing allows for the creation of bespoke implants tailored to a patient’s unique anatomical features. This personalized approach improves the compatibility and effectiveness of implants, contributing to better surgical outcomes [19-20]. Additionally, the ability to rehearse surgeries on 3D models represents a monumental step forward. Surgeons can simulate the entire procedure, identify and address potential hurdles, and refine their techniques before entering the operating room. This rehearsal not only boosts surgeon confidence but also significantly reduces the risk of intraoperative errors, enhancing overall surgical safety and efficacy.

The integration of 3D printing technology into cervical laminoplasty training modules signifies a paradigm shift in surgical education and practice. By transforming digital imaging data into tangible, patient-specific models, this technology bridges the gap between abstract imaging and real-world application. The tactile and visual insights provided by these models enrich the learning experience, making it as close to actual surgery as possible without the associated risks. Beyond training, 3D models also play a key role in improving patient-doctor interactions. They allow surgeons to explain complex procedures in an accessible and visual manner, fostering patient understanding and trust. This transparency enhances shared decision-making and aligns patient expectations with clinical realities [10]. However, this applies only when the 3D model is based on the CT scan of the patient to be treated, ensuring an accurate representation of their unique anatomy. Generic 3D models, while useful for general education, lack the specificity needed for precise preoperative planning and personalized patient communication [13].

This study aims to evaluate the perceived educational value of 3D-printed cervical laminoplasty models among the participants. Specifically, it seeks to (1) assess participants’ perceptions regarding the realism and anatomical accuracy of the models; (2) evaluate the perceived impact of 3D models on understanding cervical laminoplasty techniques; (3) determine whether participants believe these models improve surgical planning and patient communication; and (4) identify potential limitations and areas for improvement in the use of 3D models for neurosurgical training.

## Materials and methods

Study design and participants

This prospective educational intervention study was conducted during the Spine Laminoplasty Course in Tyumen, Russian Federation, in September 2023. The primary aim of the study was to assess the effectiveness of 3D-printed cervical vertebrae models in enhancing participants' learning experiences and surgical skills. The study included 38 participants comprising neurosurgeons, neurosurgery residents, medical students, and other healthcare professionals. All participants actively engaged in the course activities and contributed to the study evaluation.

Informed consent and confidentiality

Medical imaging data from a single volunteer were used to create the 3D models. Informed consent and confidentiality were critical to maintaining the study’s ethical integrity. The volunteer was thoroughly briefed on the study’s objectives, potential risks, and benefits, ensuring voluntary participation with the right to withdraw at any time. To safeguard the volunteer’s sensitive information, stringent confidentiality protocols were enforced. Any deviations or issues related to consent or confidentiality were promptly addressed and documented to uphold the study's ethical standards.

3D model creation

**Figure 1:**
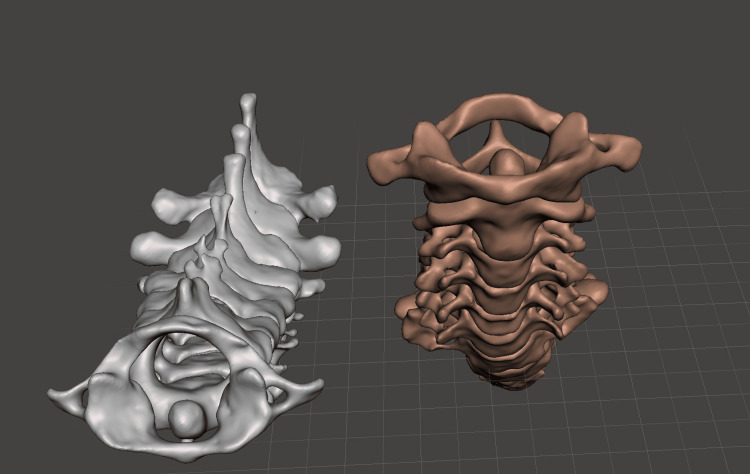
The digital processing of the anatomical models included creating anterior and superior views, during which the vertebral body was fused, and a stable base was designed to en-sure the model's stability during the workshop This processing was performed using Meshmixer® software, enabling precise modifications to enhance the model's usability and structural integrity

**Figure 2:**
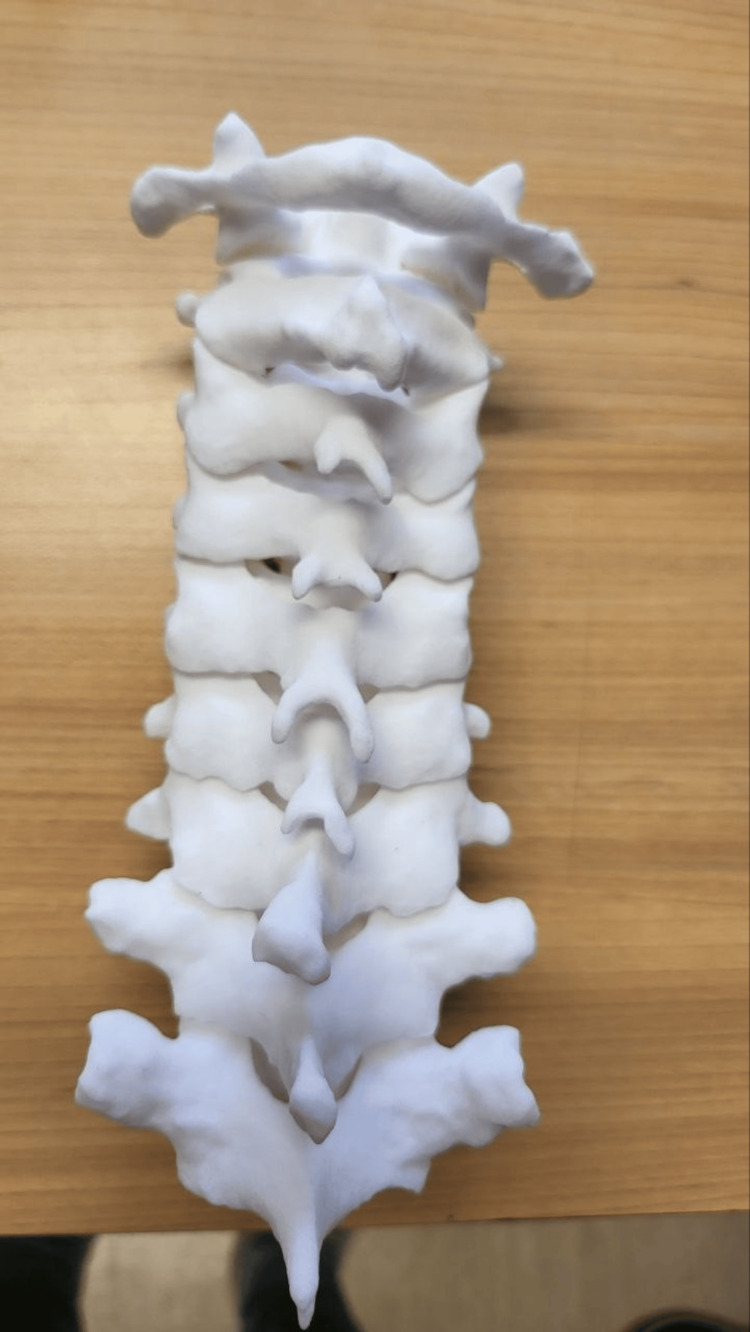
Superior view of a 1:1 scale 3D-printed anatomical model from C1 to T2

The data was acquired from a high-resolution CT scan using the Canon Aquilion One system. The resulting Digital Imaging and Communications in Medicine (DICOM) data were processed using Horos®, a free software platform [19]. Horos® utilizes the Hounsfield unit threshold (cortical bone has a high value) to generate a 3D vectorial model. While this mesh achieves high accuracy, minor artifacts may still be present. These artifacts were addressed by exporting the model in .obj format and refining it using Meshmixer® 3.5 (Autodesk Inc., San Rafael, CA) [20] (Figure 1). The refined 3D model was then imported into Cura® 4.6 software (Ultimaker, Geldermalsen, The Netherlands) [21], where printing parameters were configured and the model was exported in .gcode format. This format allowed the FFF 3D printer (Anet A8®, Anet Technology Co., Shenzhen, China) to interpret the instructions and deposit the fused material to create the skull models with precision (Figure 2).

3D printing

With the models finalized and slicing completed, the 3D printing process was initiated. Polylactic acid (PLA) filament was loaded into the printer, and the printing was conducted under carefully monitored conditions to ensure optimal accuracy and quality. Printing parameters were meticulously adjusted as needed to achieve the desired precision, ensuring that the physical model accurately represented the digital design.

Post-processing

**Figure 3:**
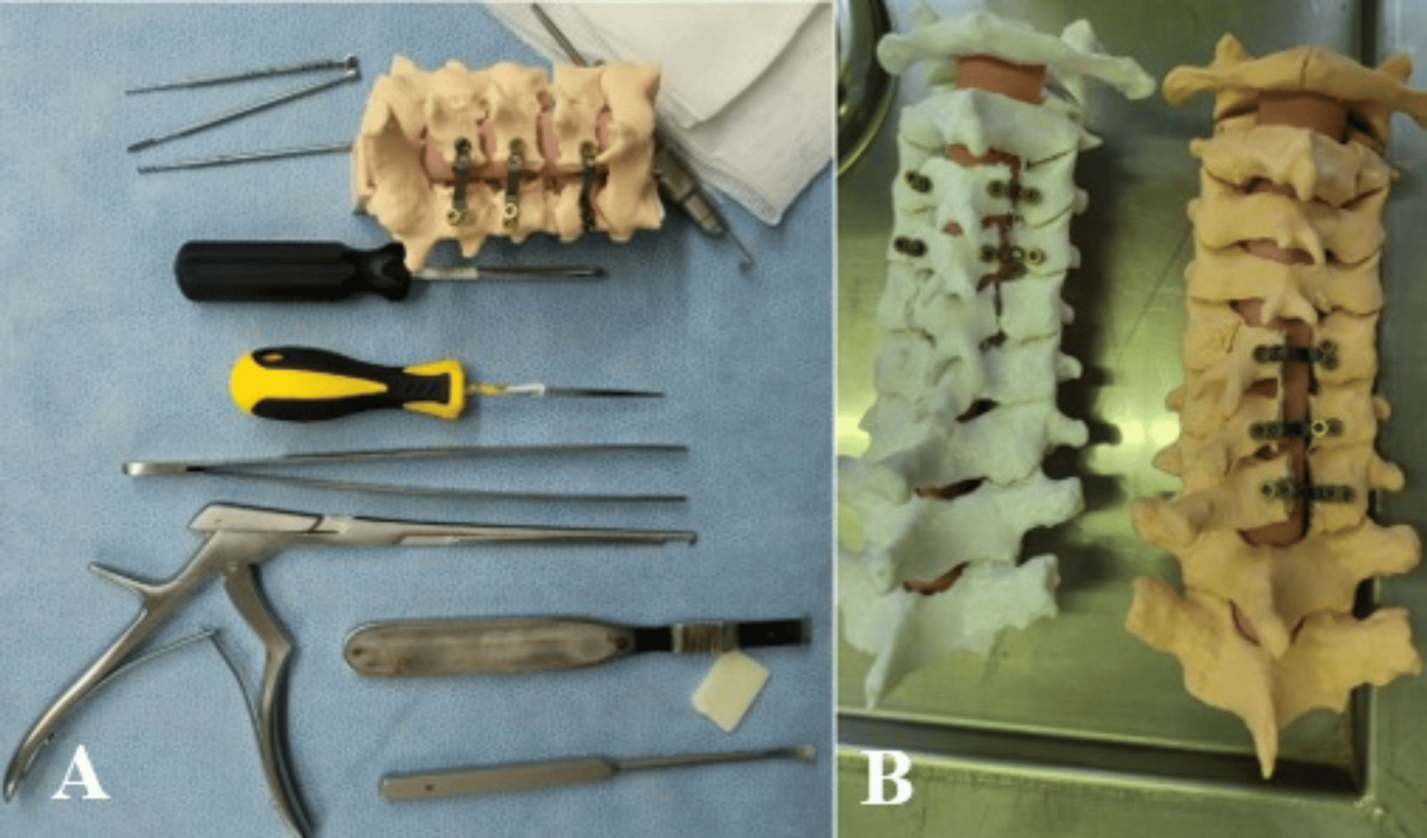
(A) A dorsolateral view showcasing the osseous structures following C3–C6 laminoplasty, highlighting the spatial configuration and anatomical modifications post-procedure. (B) A superior view presenting the osseous structures after targeted laminoplasty procedures at C3–C4 and C5–C7, illustrating the structural changes and expanded space achieved in each region

Following the printing phase, the models underwent comprehensive post-processing steps. These included the removal of support structures, smoothening of rough surfaces, and fine-tuning to ensure the models were ready for practical application. Each model was meticulously inspected to confirm its accuracy and fidelity to the original digital designs and imaging data. This quality assurance process ensured the models were suitable for deployment in surgical planning and educational scenarios (Figure 3).

Course structure

The Spine Laminoplasty Course integrated a comprehensive curriculum that included theoretical lectures, live surgical demonstrations, and interactive hands-on training sessions utilizing 3D-printed models. Participants had the opportunity to perform cervical laminoplasty procedures on these models under the direct supervision and guidance of experienced spine surgeons, fostering a practical and immersive learning experience (Figure 3).

Survey development

Following the course, a survey was conducted to assess participants' perceptions and experiences regarding the effectiveness of the 3D-printed models in enhancing their understanding and surgical skills. The survey included a series of targeted questions evaluating key aspects such as the realism of the models, the value of the hands-on training, and the participants' perceived improvement in surgical proficiency (Table 1) [21].

**Table 1:** Feedback survey on 3D-printed laminoplasty models

	Personal information
	Profession
Section 2: Experience with 3D-printed models	
	Have you used 3D-printed models in a medical setting prior to this course?
	How useful did you find the 3D-printed laminoplasty models in understanding myelopathy and laminoplasty concepts?
	Do you think the 3D-printed models enhanced the learning experience compared to traditional teaching methods?
Section 3: Overall feedback	
	Would you recommend the use of 3D-printed models in other medical courses or practices?
Section 4: Application of 3D models in neurosurgery and spinal procedures	
	How accurately do you think the 3D-printed models represent anatomical structures involved in laminoplasty?
	In your opinion, did the 3D models help in better visualizing the surgical steps for laminoplasty?
	Do you think 3D models can be a valuable tool for patient education in spinal procedures?
	How beneficial were the 3D models in planning the surgical approach for laminoplasty?
	Did the 3D models assist in identifying potential challenges or complications that might arise during surgery?
	How do you rate the quality and detail of the 3D-printed models used during the course?
	Would you prefer to use 3D models for preoperative planning in future spinal procedures?
	Do you think 3D models could replace cadaver models in neurosurgical training?
	In your opinion, what is the most significant advantage of using 3D models in neurosurgery education and practice?
	What improvements, if any, would you suggest for the 3D models used in neurosurgical and spinal education?
Section 5: Further insights on 3D models in neurosurgical education	
	How did the 3D models impact your confidence in understanding laminoplasty procedures?
	How would you rate the ease of interaction with the 3D-printed models?
	Do you think the 3D models provided a realistic representation of spinal anatomy?
	How useful were the 3D models in explaining surgical procedures to patients?
	Did the 3D models help in improving the communication among the medical team?
	What type of 3D models do you find most beneficial for your practice?
	Do you think 3D printing technology should be integrated more extensively in medical education and practice?
	Were there any challenges or limitations you faced while using the 3D models? Please specify
	How likely are you to use 3D models in your future practice?
	Do you think using 3D models can shorten the learning curve for understanding complex spinal procedures?

Survey administration

The survey was distributed to all participants immediately after the conclusion of the course. Participation was voluntary, and responses were collected anonymously to ensure privacy and encourage honest feedback.

Data analysis

The survey responses were analyzed using descriptive statistics to evaluate the participants' overall perceptions of the 3D models. The analysis emphasized the models' impact on enhancing surgical skills and boosting confidence, providing insights into their effectiveness as a training tool.

Ethical consideration

The study was conducted in compliance with the ethical standards set by the institutional research committee and aligned with the 1964 Helsinki Declaration and its subsequent amendments. Informed consent was obtained from all participants involved in the study to ensure ethical integrity and voluntary participation.

Confidentiality

All data were managed with strict confidentiality, and participant anonymity was upheld throughout the study. This structured approach aimed to explore and highlight the potential advantages of incorporating 3D printing technology into surgical education, with a particular focus on enhancing training in cervical laminoplasty.

## Results

**Figure 4:**
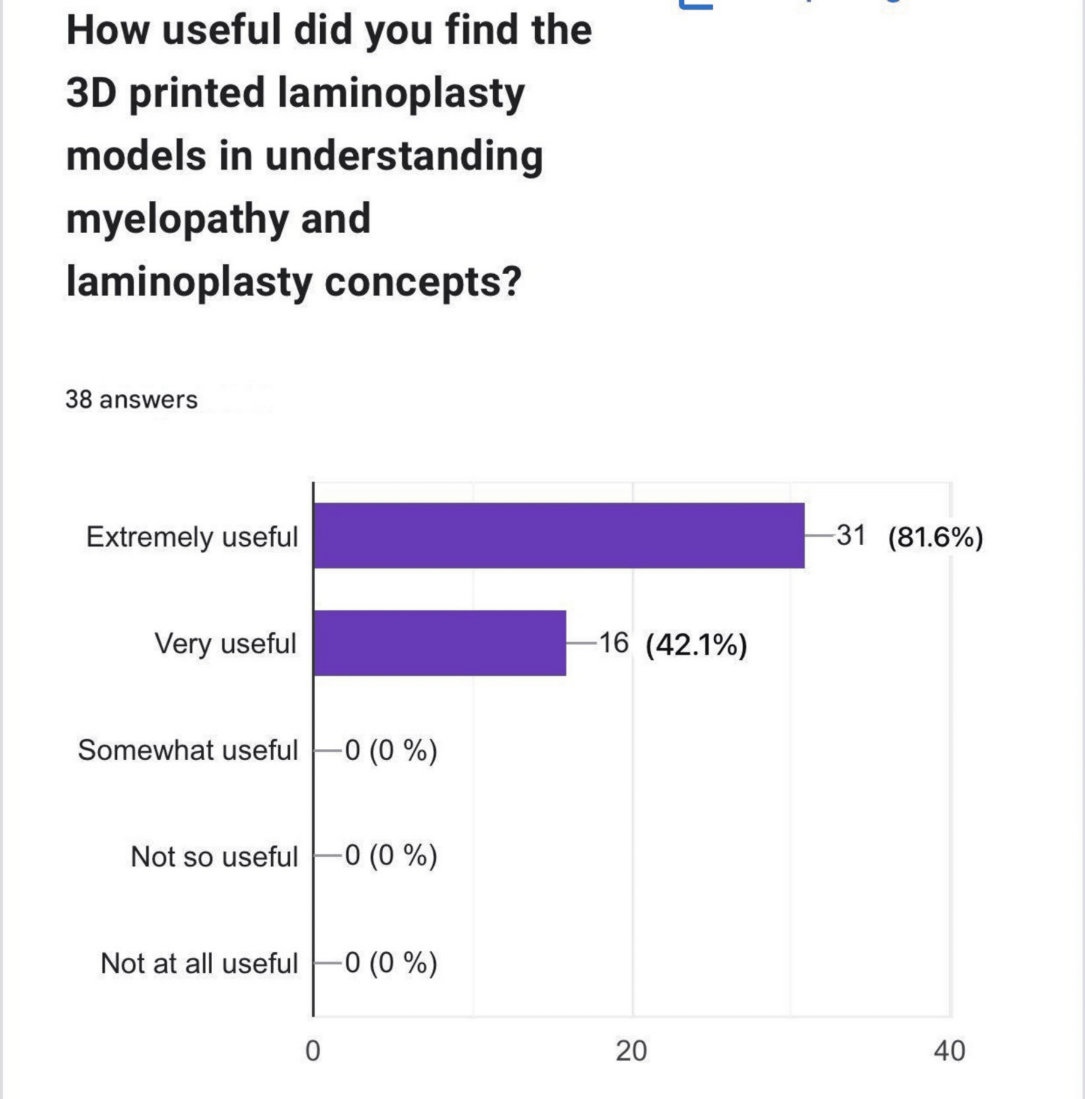
Perception of usefulness

The feedback survey on the 3D-printed laminoplasty models provided valuable insights into their effectiveness as an educational tool in neurosurgery and spinal procedures, particularly for understanding key concepts related to myelopathy and laminoplasty. A significant majority of respondents found the models highly beneficial, with 31 (81.6%) rating them as "extremely useful" and 16 (42.1%) as "very useful." Notably, no participants rated the models as "less useful," underscoring their widespread acceptance and value in educational settings (Figure 4).

**Figure 5:**
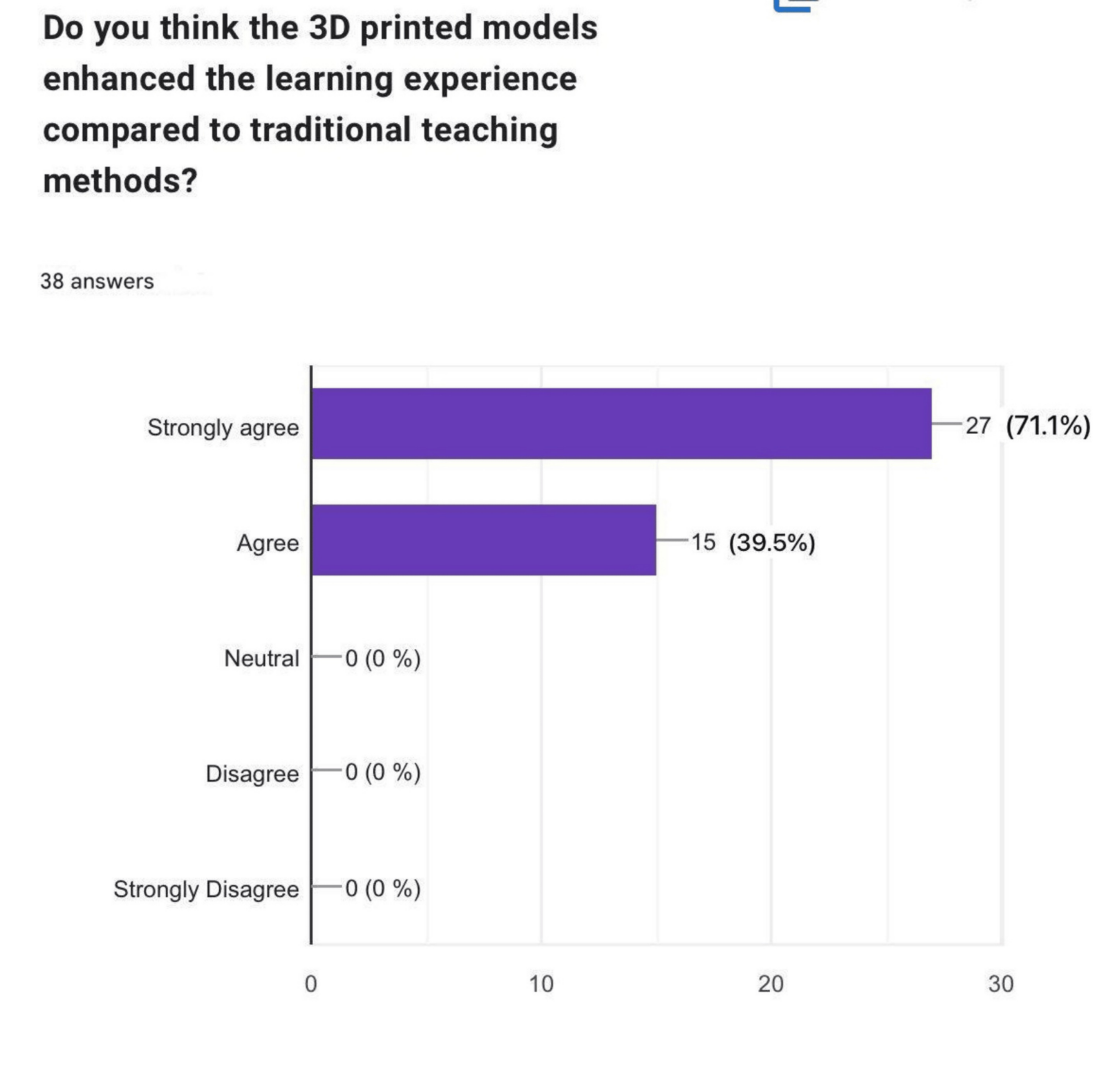
Enhanced learning experience

When comparing the 3D-printed models to traditional teaching methods, a significant proportion of participants acknowledged the enhanced learning experience provided by the models. Specifically, 27 (71.1%) "strongly agreed," and 15 (39.5%) "agreed" that the models contributed positively to their understanding. Notably, there were no neutral or negative responses, emphasizing their effectiveness as an educational tool (Figure 5).

**Figure 6:**
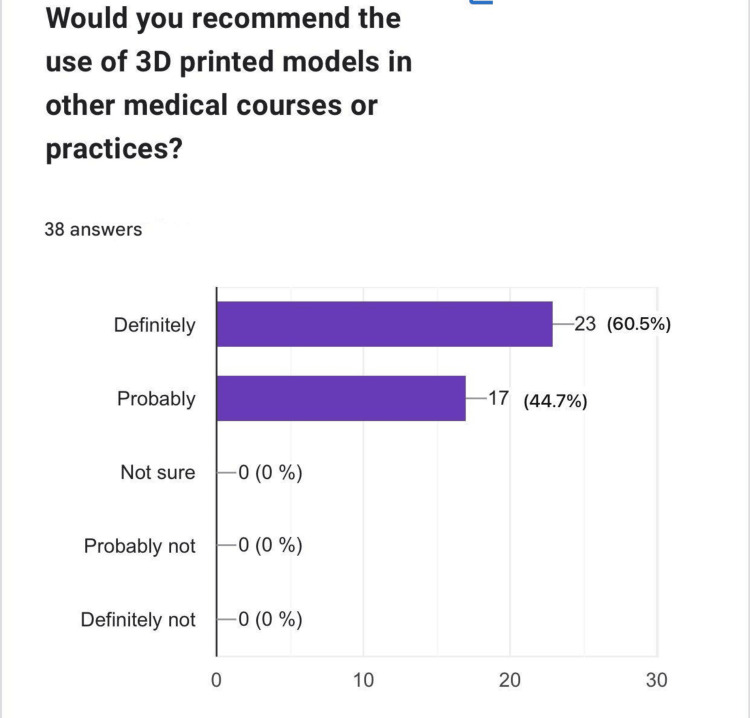
Recommendation for broader use

The participants' enthusiasm for the 3D-printed models was further evident in their willingness to recommend their use in other medical courses or practices. Of note, 23 (60.5%) respondents indicated they would "definitely" recommend the models, while 17 (44.7%) stated they would "probably" recommend them, underscoring their perceived value in medical education (Figure 6).

**Figure 7:**
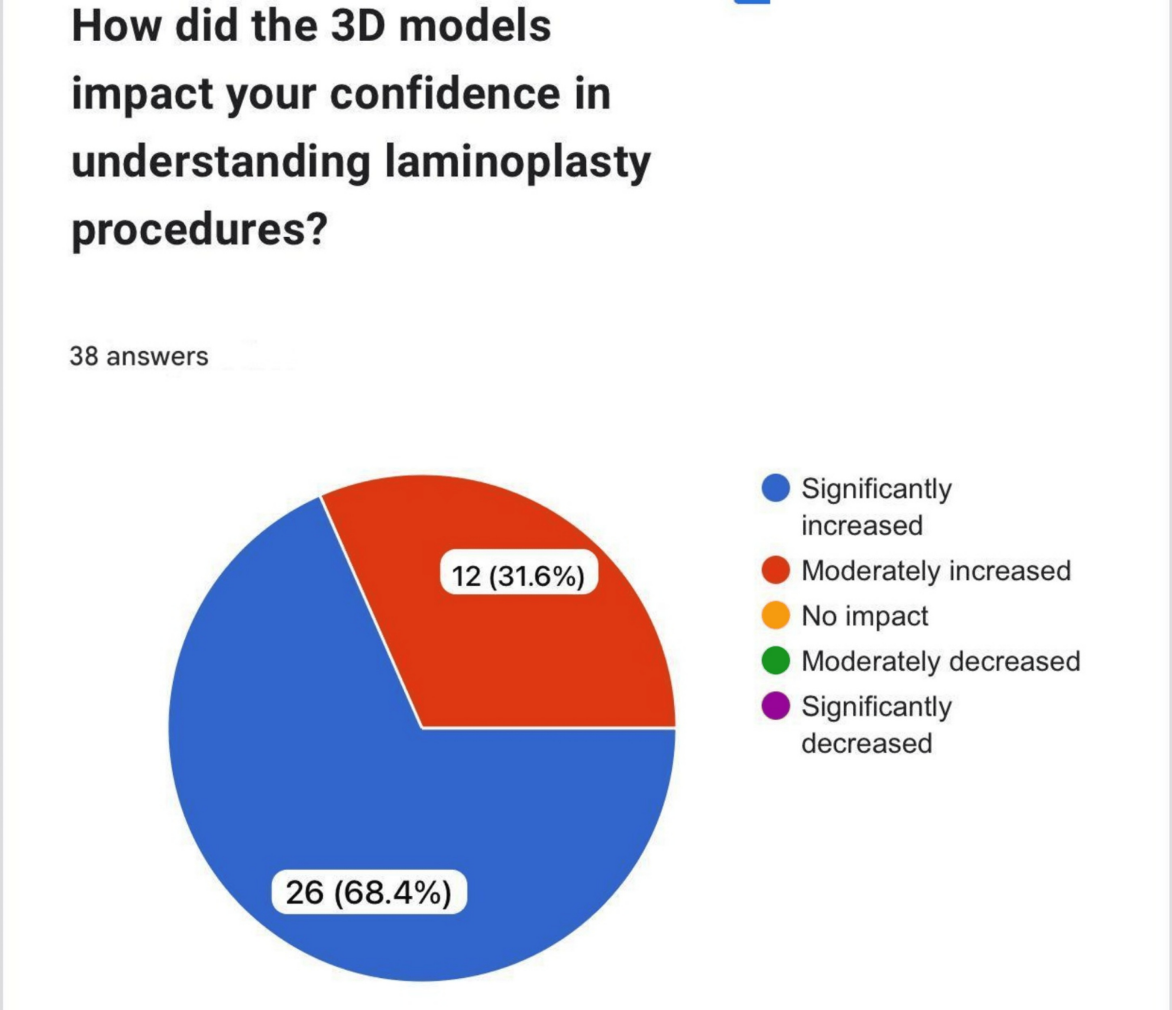
Confidence and ease of use

The use of 3D models significantly boosted participants' confidence in understanding laminoplasty procedures. Among the respondents, 26 (68.4%) reported a "significant increase" in confidence, while another 12 (31.6%) noted a "moderate increase" (Figure 7).

**Figure 8:**
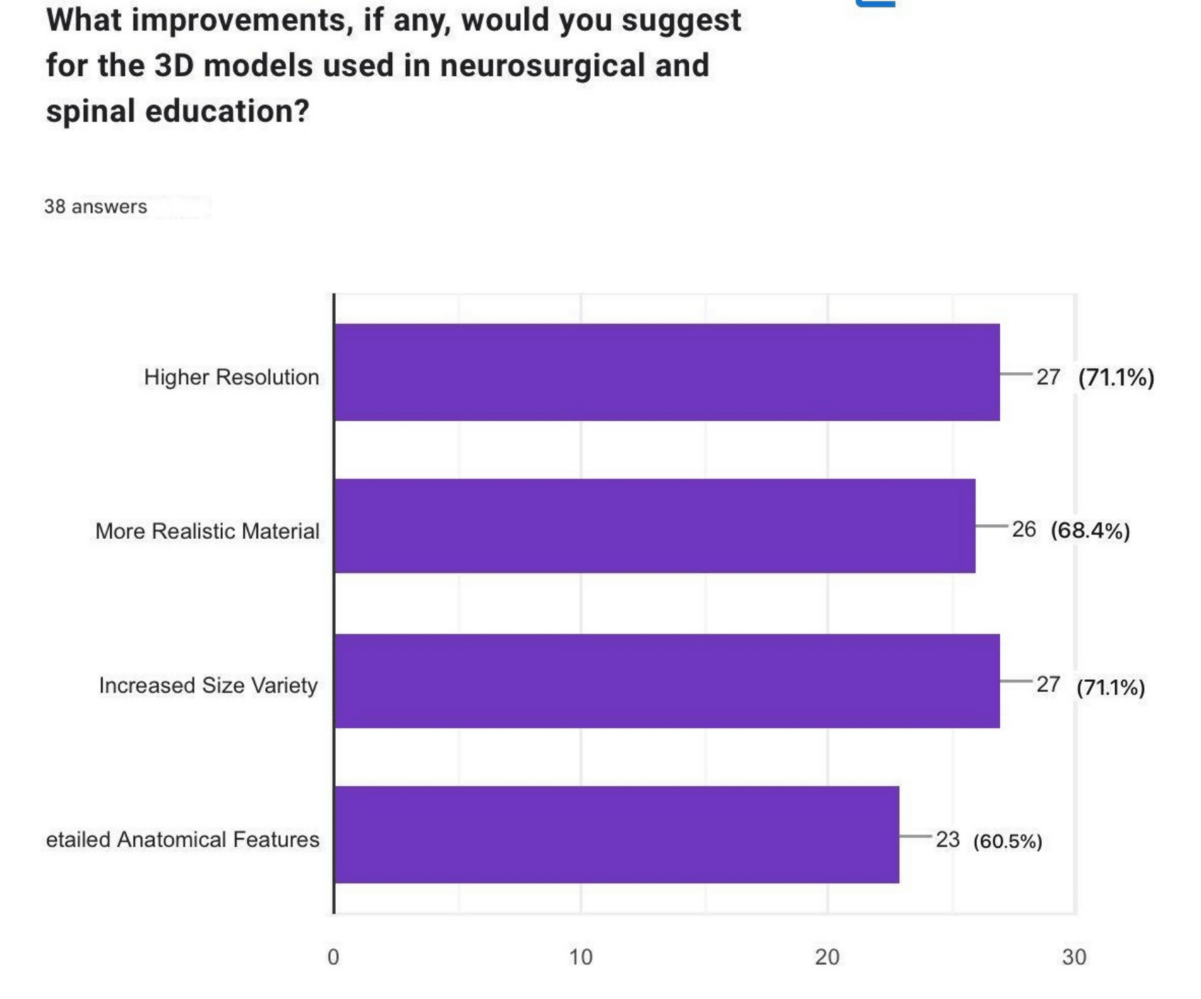
Suggestions for improvement

Participants suggested several areas for improvement in 3D-printed models, with the most common being "higher resolution" (n=27, 71.1%), "increased size variety" (n=27, 71.1%), and "more realistic material" (n=26, 68.4%). These improvements highlight the need for enhanced model realism in future developments (Figure 8).

**Figure 9:**
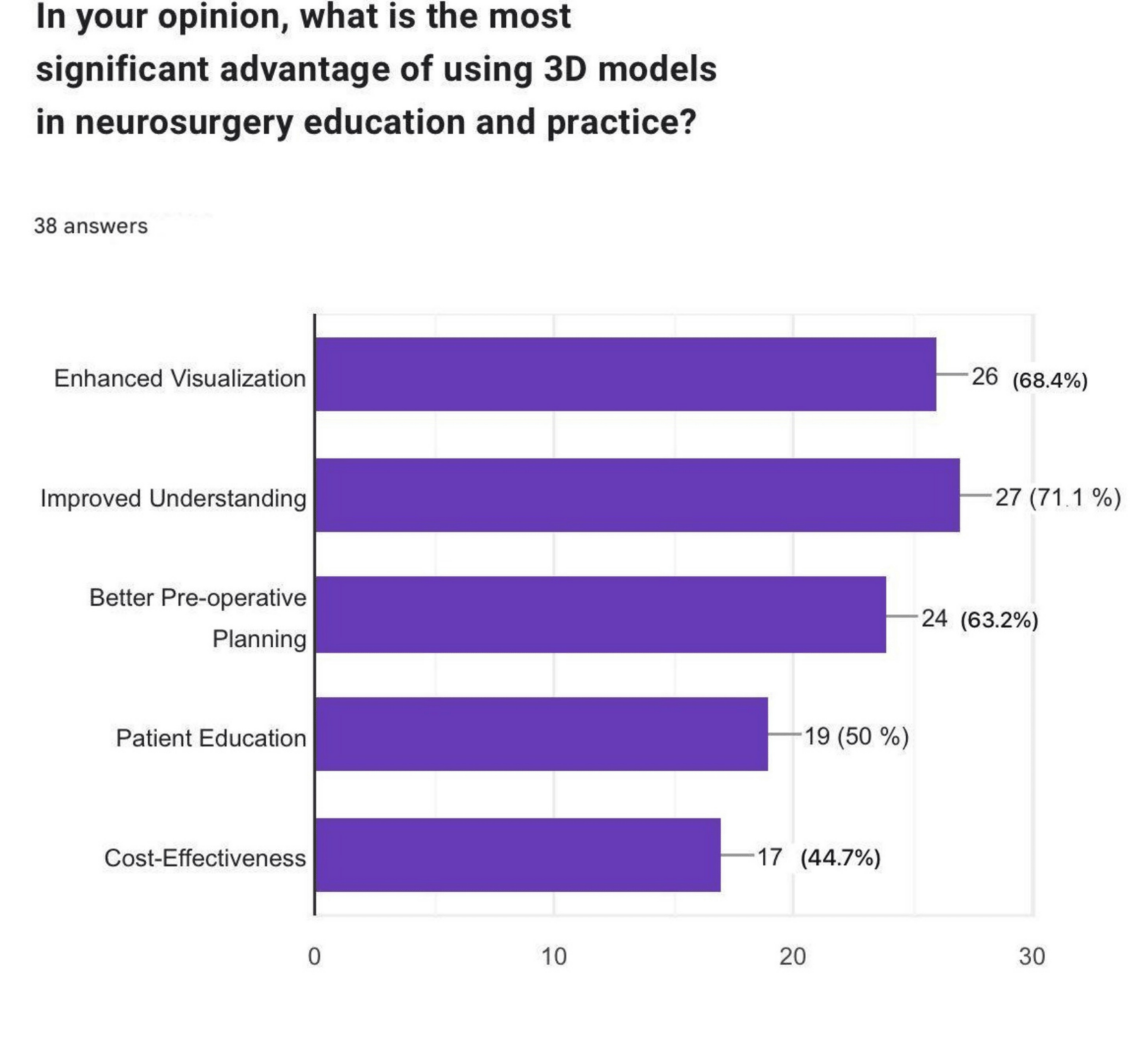
Advantages of 3D models

When asked about the most significant advantage of 3D models in neurosurgical education, participants cited "improved understanding" (n=27, 71.1%), "enhanced visualization" (n=26, 68.4%), and "better preoperative planning" (n=24, 63.2%) as the top benefits. Interestingly, "cost-effectiveness" was the least cited advantage (n=17, 44.7%) (Figure 9).

**Figure 10:**
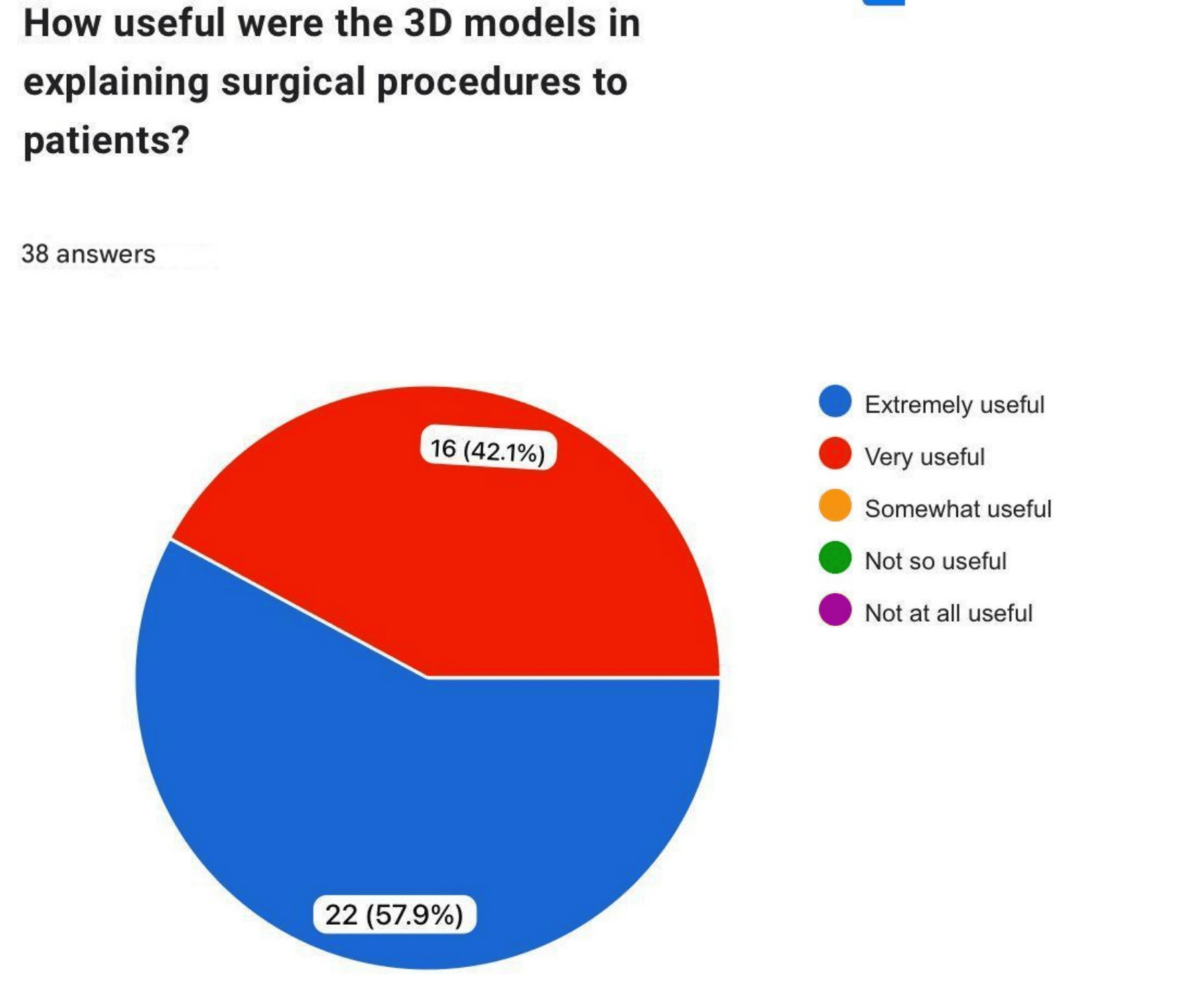
Application in patient education

When asked about the value of 3D models for patient education, a majority (n=22, 57.9%) "agreed," while 16 (42.1%) "strongly agreed" that the models could serve as an important tool for explaining spinal procedures to patients (Figure 10).

The participant pool primarily comprised neurosurgeons (n=22, 57.9%) and neurosurgery residents (n=11, 28.9%), with 89.5% having prior experience using 3D models. This prior exposure likely influenced perceptions of the models' utility and effectiveness, potentially limiting the generalizability of the findings to those with minimal or no experience using 3D models.

## Discussion

This study sought to evaluate the potential of 3D-printed models as a transformative tool in enhancing understanding and practical engagement in laminoplasty and neurosurgical education. The participants' feedback highlighted a strong consensus regarding the utility of these models in bridging theoretical knowledge and practical expertise. Survey results revealed widespread endorsement of the 3D models, with 89.5% of participants reporting improved visualization of surgical steps. This underscores the critical role of 3D models in overcoming the limitations of traditional, theory-based learning and fostering a more intuitive grasp of myelopathy and laminoplasty concepts.

The benefits of 3D models extend beyond training, demonstrating substantial utility in surgical planning. Among participants, 55.3% found the models highly beneficial for surgical planning, with 73.7% identifying them as the most advantageous application. This highlights the potential of 3D models as indispensable tools in pre-operative planning, contributing to improved surgical precision and outcomes. Additionally, 83.8% of participants affirmed the effectiveness of 3D models in explaining surgical procedures to patients, emphasizing their value in enhancing patient education and engagement. By fostering a clearer understanding of complex procedures, these models enable more informed and collaborative patient-doctor relationships.

The data also reflects improvements in inter-team communication, with 86.9% of participants reporting enhanced collaboration. Moreover, 89.4% noted increased confidence in understanding laminoplasty procedures, underscoring the broader benefits of 3D models in cultivating a competent and cohesive medical team. A notable 81.5% of participants agreed that 3D models accelerate the learning curve, illustrating their pivotal role in facilitating rapid skill acquisition and competency development in the demanding field of neurosurgery. These findings underscore the transformative impact of 3D models across multiple dimensions of surgical education and practice.

Enhancement in visualization and understanding

The majority of respondents agreed or strongly agreed that 3D models enhance the visualization of surgical steps in laminoplasty: The procedure typically follows these stages: (1) midline or lateral exposure of the posterior cervical elements, (2) identification of the appropriate laminae and hinge sites, (3) controlled osteotomy or hinge creation using a high-speed drill or ultrasonic bone scalpel, (4) gradual elevation of the lamina to decompress the spinal cord, (5) placement of spacers or bone grafts to maintain the expanded canal, and (6) fixation with mini-plates, sutures, or other stabilization techniques, with an impressive 89.5% acknowledging their contribution to improved understanding and procedural clarity. This finding supports the hypothesis that the detailed and three-dimensional perspectives offered by these models are instrumental in aiding the conceptualization and retention of complex anatomical structures and surgical procedures.

3D models were widely recognized as an effective tool for patient education, with nearly 89.4% of participants agreeing or strongly agreeing on their value. Additionally, 83.8% found these models extremely or very useful in explaining surgical procedures to patients. By enhancing the communicative process, 3D models bridge the knowledge gap between medical professionals and patients, fostering a collaborative environment that promotes informed decision-making and improved patient outcomes [22-24]. Moreover, a significant majority reported that these models improved communication among medical team members. This enhanced inter-team communication is critical for achieving coordinated and effective patient care, underscoring the multifaceted benefits of integrating 3D models into clinical practice [25].

Preoperative planning and identification of complications

The unanimous recognition of the benefits of 3D models in planning surgical approaches and identifying potential complications highlights their practical utility in clinical settings. By enabling surgeons to anticipate challenges, these models facilitate meticulous pre-operative planning, which can reduce intraoperative risks and enhance surgical outcomes [26-29]. While 91.8% of participants rated the quality and detail of the 3D models as excellent or very good, several suggestions for improvement emerged. These included higher resolution, the use of more realistic materials, increased size variety, and greater anatomical detail. Implementing these enhancements could further elevate the learning experience, providing an even more accurate representation of anatomical structures and reducing reliance on traditional learning tools such as cadaver models [30-32].

3D models in medical training

The data revealed a slight divergence in opinion regarding the complete replacement of cadaver models with 3D models in neurosurgical training. Nonetheless, the acknowledged benefits of 3D models in surgical planning, procedure rehearsal, and the creation of patient-specific models underscore their versatility and adaptability in various educational and clinical scenarios.

Cadaveric dissection remains a valuable tool in surgical training, enabling surgeons to learn regional anatomy, gain familiarity with instruments, and develop essential manual skills [33-36]. However, the challenges associated with cadaver use, such as high costs, legal restrictions, and the need for specialized facilities for preservation and storage, pose significant barriers, particularly in low-income countries and non-university hospital settings [37-39].

The COVID-19 pandemic further highlighted the vulnerabilities of cadaver-based training, with practices significantly curtailed due to health and logistical concerns [13,14]. In such circumstances, 3D printing offers a practical alternative. It allows the creation of multiple models from a single patient dataset, enabling reproducible and scalable training opportunities. This capability supports widespread use without the ethical, logistical, or financial challenges of cadaver acquisition and maintenance.

Despite its advantages, 3D printing is not without limitations. Materials suitable for printing are constrained by their thermodynamic properties, and the production of complex or large-scale models can be time-intensive and prone to printing defects [31]. However, rapid prototyping offers a significant advantage by quickly generating 3D models from medical imaging data. These models provide surgeons with both 2D visual information and tactile feedback, offering a comprehensive training and planning tool [32].

Impact on confidence and learning curve

3D models significantly boosted confidence in understanding laminoplasty procedures for 89.4% of participants. Additionally, these models show great potential in shortening the learning curve for mastering complex spinal procedures. While participants perceived them as beneficial for learning, the study did not objectively evaluate improvements in skill acquisition or procedural competency. Further research with performance-based assessments is required to determine their true impact on shortening the learning curve and enhancing surgical proficiency.

Integration into medical education and practice

There is unanimous agreement among participants on the importance of integrating 3D printing technology more extensively into medical education and practice, with 100% expressing a likelihood to incorporate 3D models into their future work. This consensus highlights the transformative role of 3D models as essential tools in modern medical training and clinical care, shaping the future of medical education and patient care methodologies [36].

Fused filament fabrication (FFF) is a widely accessible 3D printing technology, with PLA being a commonly used material. PLA offers several advantages, including non-toxicity and sustainability, as it is derived from the bacterial fermentation of carbohydrates such as corn, cassava, and carp. It is not oil-based and undergoes low-temperature sterilization, typically using 100% ethylene oxide, which preserves its physical and anatomical properties [16,35].

However, PLA also has limitations. Its low crystallization (55 °C) and melting temperatures (180 °C) require careful handling during procedures such as drilling. Using a low-speed drill is essential to prevent material melting, ensuring the integrity of the model during use [35]. Despite these drawbacks, PLA remains a versatile and practical material for 3D printing in medical applications.

Limitations of the study

The study acknowledges inherent limitations in the 3D printing processes used to create anatomical models. These limitations can substantially impact the models' quality, accuracy, and utility, potentially affecting the learning experience and outcomes for the participants. Here are the detailed points regarding the printing limitations:

Material Limitations

The 3D models are created using PLA, a material that, while practical and widely used, does not accurately replicate the properties, texture, and feel of biological tissues. This limitation can affect the realism and overall learning experience. Additionally, the thermodynamic constraints of PLA restrict the range and complexity of models that can be produced, potentially limiting its applicability in creating diverse anatomical simulations.

The participant pool, primarily composed of neurosurgeons and neurosurgery residents, may introduce a potential bias. Their more advanced expertise and perspectives might overshadow the viewpoints and learning experiences of medical students and neurology participants, who constituted a smaller percentage of the cohort (3.9% and 9.3%, respectively). This imbalance could influence the generalizability of the study’s findings across broader educational levels and specialties.

## Conclusions

The integration of 3D-printed models into cervical laminoplasty training represents a significant leap forward in medical education, marking a transformative shift from traditional learning methods. The unanimous endorsement from participants highlights the enhanced learning experience, improved visualization, and deeper understanding facilitated by these models. Beyond education, 3D models have shown substantial promise in preoperative planning and risk mitigation, proving instrumental in patient education and fostering better inter-professional communication. These advantages collectively contribute to improved patient outcomes and strengthened team collaboration. While participants widely praised the models for their quality and accuracy, suggestions for improvements in resolution and anatomical detail indicate areas where 3D modeling technology could further evolve. Although there were reservations about fully replacing cadaver models, the adaptability and versatility of 3D models were overwhelmingly recognized. This underscores their indispensable role in modern medical training and practice.

This study demonstrates the transformative potential of 3D-printed models, heralding a new era in medical education. These models are paving the way for more interactive, immersive, and precise learning experiences, thereby shaping the future of medical training and enhancing the overall efficacy of healthcare delivery.
